# Supplementary material for: Deep learning-based unlearning of dataset bias for MRI harmonisation and confound removal
Source: Neuroimage. 2021 Mar;228:117689. doi: 10.1016/j.neuroimage.2020.117689 (PMC7903160; doi:10.1016/j.neuroimage.2020.117689)
Supplement: Supplementary Data S1 — Supplementary Raw Research Data. This is open data under the CC BY license http://creativecommons.org/licenses/by/4.0/ [file mmc1.pdf]

10. Supplementary Materials

10.1. Training Graphs

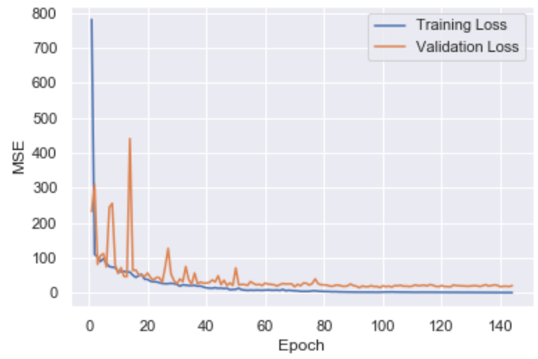

(a) Main Task Loss

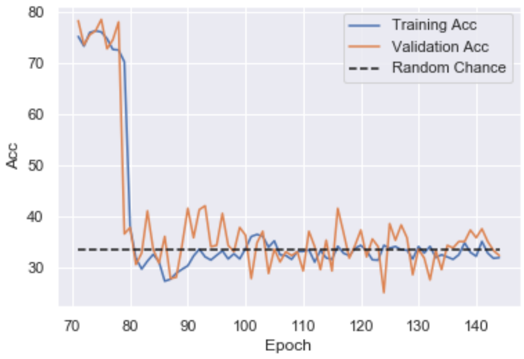

(b) Scanner Classification Accuracy

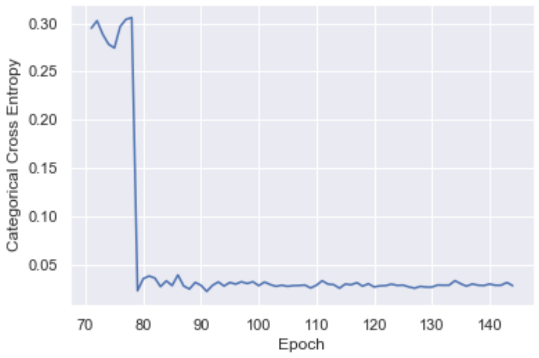

(c) Domain Loss - Training

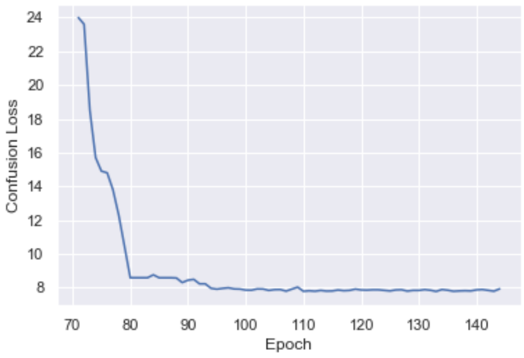

(d) Confusion Loss - Training

Figure 14: Graphs for the different loss functions from training. Only the main task loss is used during pretraining and so all other losses begin at 70 epochs when convergence on the main task is achieved.

Example training graphs from the age prediction task with three datasets. Only the main task loss (MSE for age prediction) is used during pretraining and so the other loss functions begin at epoch 70 when unlearning started. Unlearning began when convergence was achieved on the main task (patience = 15 epochs). It can be seen that beginning unlearning does not reduce the performance on the main task. It can also be seen that all the loss functions are stable throughout training.

10.2. DANN Results

A DANN-style domain adaptation approach was also tried due to its simpler implementation. As confirmed across the literature, it was unstable to train, limiting its performance. The results from training can be see in Table 8 where it can be seen that the scanner classification accuracy was much lower than random chance, indicating that scanner information remained in the feature representation. It can also be seen that the performance on the main task was reduced, with the domain adaptation leading to lower performance compared to normal training.

Training Data			Biobank MAE	OASIS MAE	Scanner Classification Accuracy %
	B	O			
**Normal Training**					
1.	✓	×	3.25 ± 2.36	16.50 ± 6.77	-
2.	×	✓	5.61 ± 3.52	4.27 ± 3.79	-
3.	✓	✓	3.30 ± 2.50	4.00 ± 2.78	98 (50)
**DANN**					
4.	✓	✓	3.35 ± 5.53	4.21 ± 6.02	7.2 (50)

Table 8: Comparing normal training on the different combinations of datasets to using DANN to remove scanner information.

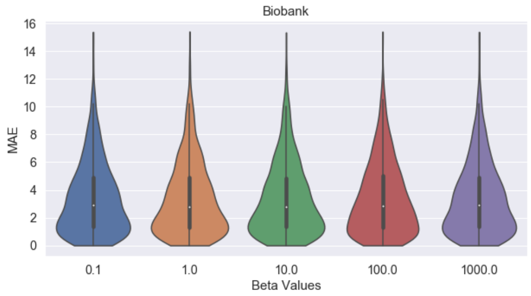

(a) Biobank MAEs

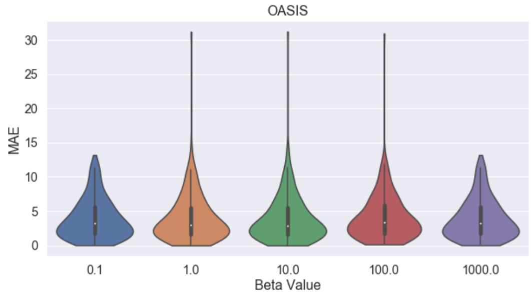

(b) OASIS MAEs

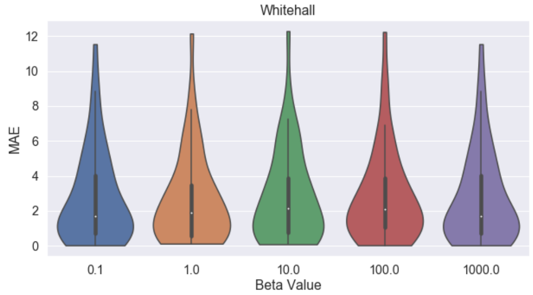

(c) Whitehall MAEs

Figure 15: MAEs for each dataset with increasing values of the constant β used in training, where β is the weight determining the contribution of the confusion loss.

10.3. Effect of Hyper-Parameters

For all experiments, the value of α remained set to 1 and no change was required. The value of β was varied between experiments, controlling the training stability and the rate of convergence: higher β values provide more stability for the domain unlearning but decrease the rate of convergence for the main task. It should not have any effect on the final age prediction values achieved, given that for all values we are able to reach convergence, and so we varied the value of β between 0.1 and 1000 and compared the MAEs for the three datasets. It can be seen that the value of β has no effect on the achieved MAEs across these values from Fig. 15. Therefore, the segmentation performance is robust to the choice of β and the value chosen can be selected to maximise the stability of training without impacting on the final prediction values. Stability could also be controlled by using different learning rates for each stage; however, this was found to be harder to tune in practice.

Finally, we explored the choice of the batch size used. For training, the largest batch that could fit into memory was used to obtain the results in the paper; here the effect of smaller batches is explored. Figure 16 shows the validation loss during training for batch sizes of 3, 8 and 32 (other batch sizes are not shown for clarity) and the pattern was the same across the different loss functions. It can be seen that the training was more stable with the smaller batch sizes. However, it can be seen from Fig. 17 that the overall MAEs achieved across the datasets were better with larger batch sizes. This is most likely because larger batch sizes force the network to unlearn scanner information more thoroughly than,

for instance, with a batch size of 3, where given our constraint on the batches, there can only be one example per scanner. Given the result from varying the value of β above, we should select the batch size so as to maximise the performance on the main task and vary the β value to control the stability of training.

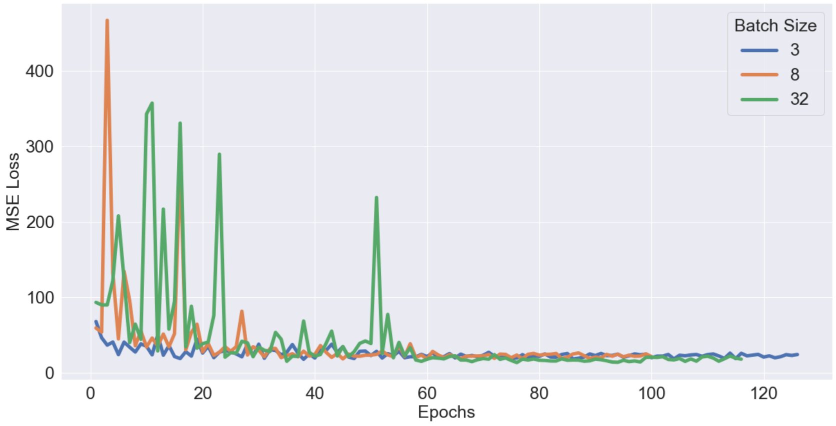

Figure 16: Validation loss with epochs for batch sizes of 3, 8 and 32.

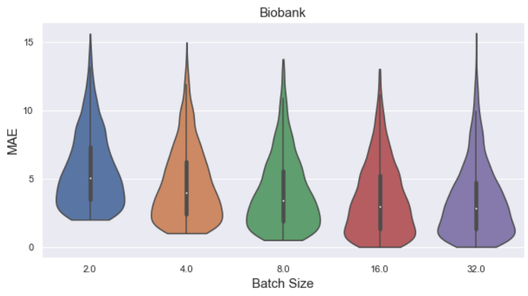

(a) Biobank MAEs

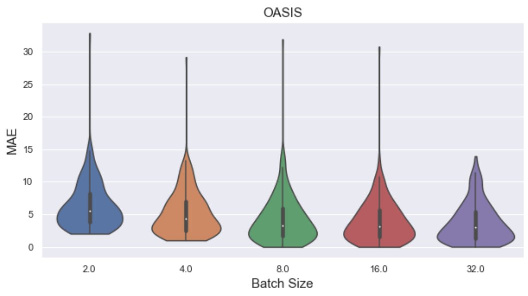

(b) OASIS MAEs

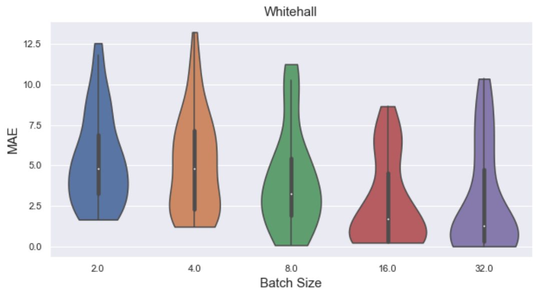

(c) Whitehall MAEs

Figure 17: MAEs for each dataset with increasing batch sizes used during training.

10.4. p Values

Paired t-tests have been completed between experiments and the uncorrected p-values are reported separately for each dataset. For each table in the results section, the rows compared are indicated in the first column of the table.

10.4.1. Age Prediction - Basic Fully-Supervised Learning

Rows Compared	Biobank	OASIS	Whitehall
4) - 9)	0.038	0.567	0.621
5) - 10)	0.094	1.63×10^{-14}	0.001
6) - 11)	1.69×10^{-14}	0.001	1.21×10^{-10}
7) - 12)	0.002	0.042	4.61×10^{-7}
4) - 13)	0.223	0.923	0.032
5) - 14)	0.099	9.42×10^{-15}	8.62×10^{-20}
6) - 15)	4.21×10^{-23}	0.012	0.008
7) - 16)	0.145	3.25×10^{-12}	1.96×10^{-22}
9) - 13)	0.416	0.004	0.070
10) - 14)	0.893	0.034	0.041
11) - 15)	3.96×10^{-24}	1.30×10^{-4}	3.61×10^{-8}
12) - 16)	0.011	0.666	0.098

1) - 16) Biobank Only: 0.103
2) - 16) OASIS Only: 5.62×10^{-31}
3) - 16) Whitehall Only: 9.71×10^{-71}

10.4.2. Age Prediction - Balanced Datasets

Rows Compared	Biobank	OASIS	Whitehall
1) - 3)	4.65×10^{-11}	2.34×10^{-18}	2.98×10^{-10}
2) - 4)	5.67×10^{-4}	1.98×10^{-3}	7.45×10^{-6}

10.4.3. Age Prediction - Biased Datasets

Rows Compared	Biobank	OASIS
1) - 2)	5.68×10^{-89}	8.62×10^{-89}
1) - 3)	2.98×10^{-92}	5.78×10^{-93}
2) - 3)	5.63×10^{-4}	0.098
4) - 5)	3.69×10^{-65}	7.14×10^{-75}
4) - 6)	3.48×10^{-72}	9.01×10^{-83}
5) - 6)	9.72×10^{-4}	0.032
7) - 8)	6.91×10^{-42}	4.78×10^{-61}
7) - 9)	9.01×10^{-51}	1.00×10^{-63}

8) - 9)	0.045	0.029

10.4.4. Age Prediction - Sex Correlated with Scanner

Rows Compared	Biobank	OASIS
1) - 2)	0.456	0.892
1) - 3)	0.416	0.493

10.4.5. Age Prediction - Sex Correlated with Age

Rows Compared	Biobank	OASIS
1) - 2)	0.001	0.009
1) - 3)	0.752	0.042

10.4.6. Segmentation - Location of Domain Classifier

Rows Compared	Biobank	OASIS
1) - 2)	9.72×10^{-4}	3.00×10^{-3}
1) - 3)	0.003	0.091

10.4.7. Segmentation - Method Comparison

Rows Compared	Biobank	OASIS
3) - 4)	0.645	0.986

1) - 4) Biobank Only: 0.833
2) - 4) OASIS Only: 0.654

10.5. Comparison to Adding Site as Regressor

Here we explored whether there were an advantage to harmonising the network compared to training a network normally and adding scanner covariates to a linear model that is used for the analysis.

To do this we considered the situation where we have data from three sites and scanners but only have manual segmentation results for a single site, which is a common scenario when working with neuroimaging data as labels are expensive to acquire. We then wanted to train a network to complete the segmentation for our test subjects from all three sites (e.g. from the ABIDE data) and then use them in a standard GLM to explore the task of interest. In this case we considered exploring the relationship between age and the normalised grey matter/white matter/CSF volumes.

We trained the model using normal training on just the labelled data (UM) and also with unsupervised unlearning, harmonising the data (main task labels for just UM but unlearning scanner information from all three sites). When we trained the model on just UM and created output predictions for the remaining two sites, we found that we had good predictions for the test data from UM but there was a significant performance drop when we applied the method to the other two sites, due to domain shift. It can be seen from the figure below that although the network trained with normal training performs well on UM (average dice = 0.891 $\pm$ 0.015) the model suffers significant performance degradation when applied to the unseen sites – MPG (average dice = 0.689 $\pm$ 0.020) and Yale (average dice = 0.627 $\pm$ 0.039). It can also be seen that this loss in performance leads to significant failures such that image-derived values such as white matter or grey matter volumes are going to be very different from the expected values. Conversely, it can be seen that the performance is greatly improved using the harmonisation method even though no manual segmentation labels for MPG or Yale were used during training: average dice = 0.891 $\pm$ 0.019 (p=4e-8) and 0.892 $\pm$ 0.009 (p=4.5e-10), respectively. This comes at little cost to the performance of the segmentation on UM (average dice = 0.883 $\pm$ 0.03, p=0.015). Clearly a covariate approach would be unable to substantially reduce these image-level errors.

To go further and verify the effect for statistical tests on derived scalar quantities, we calculated the CSF volume, normalised by total brain volume. As we expect this to be indicative of age, we split the subjects into a young group and an old group, (n=44) and we used this in a GLM to explore the ability to find group differences. We considered three scenarios:

1. "normal" input data (no unlearning) but with a design matrix that has separate covariates for the scanners (as well as a simple two group, unpaired, difference regressor – this is x1 below),
2. "unlearning" data with a simple two group (unpaired) difference design matrix,
3. base case of "normal" data and simple design matrix with no scanner-based covariates.

We compute this using the statsmodel python package. The results can be found in the figures below.

It can be seen that, as expected from the image examples, when we do nothing to correct for the scanner differences (scenario 3) we do not get a significant result (p=0.745). Equally,

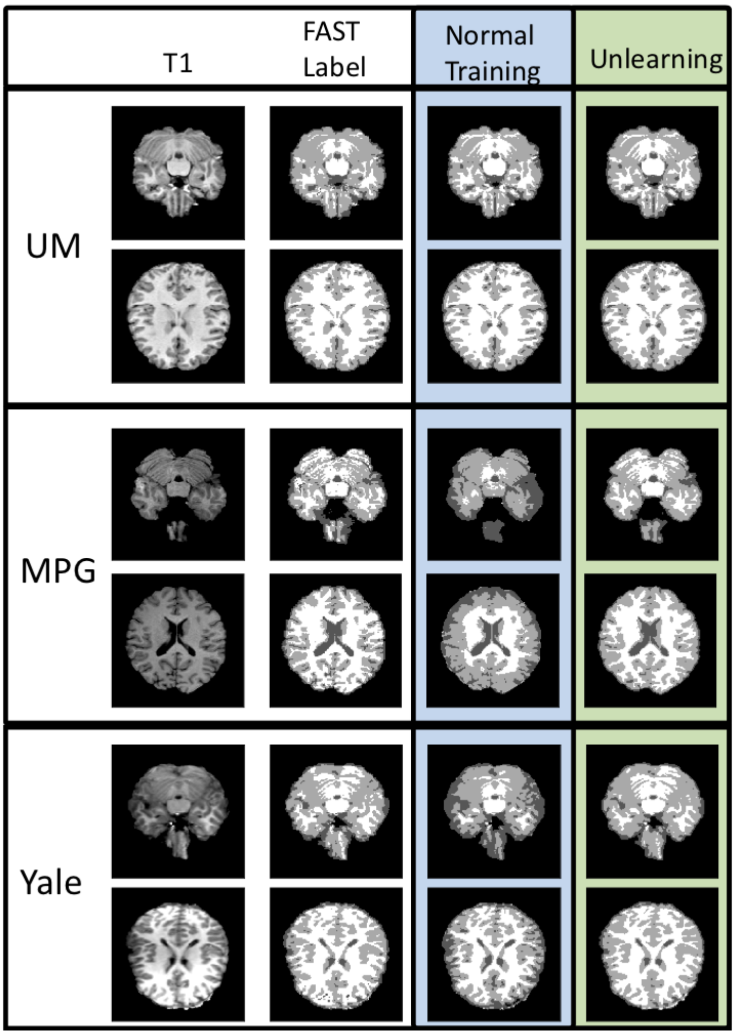

Figure 18: Segmentation results for the ABIDE data using normal training and unlearning when labels are only available for the UM site. Labels are white matter, grey matter and CSF.

when we add scanner as a regressor in the model (scenario 1) we also do not get a significant result (p=0.128) but when we use our deep learning model we get a much stronger statistic and a significant difference (p=0.009).

Therefore, there are clear scenarios in which just adding scanner-based covariates in the model leads to reduced statistical differences, in this case preventing us achieving significant results. Consequently, there is a real need for harmonisation approaches, such as this unlearning method, in situations like this.

Scenario 1:

```
                 Generalized Linear Model Regression Results
==============================================================================
Dep. Variable:                      y   No. Observations:                   44
Model:                            GLM   Df Residuals:                       40
Model Family:                Gaussian   Df Model:                            3
Link Function:               identity   Scale:                       0.0010448
Method:                          IRLS   Log-Likelihood:                 90.670
Date:                Wed, 28 Oct 2020   Deviance:                     0.041792
Time:                        11:29:27   Pearson chi2:                   0.0418
No. Iterations:                     3
Covariance Type:            nonrobust
==============================================================================
                 coef    std err          z      P>|z|      [0.025      0.975]
------------------------------------------------------------------------------
x1             0.0082      0.005      1.523      0.128      -0.002       0.019
x2             0.1446      0.007     20.013      0.000       0.130       0.159
x3             0.2467      0.010     25.393      0.000       0.228       0.266
x4             0.3048      0.010     30.467      0.000       0.285       0.324
==============================================================================
T-values:  [ 1.52273558 20.01274993 25.39284127 30.46712709]
```

(a) Scenario 1: “normal” data (no unlearning) but design matrix with covariates for the scanners (and group difference)

Scenario 2:

```
Generalized Linear Model Regression Results
==============================================================================
Dep. Variable:                      y   No. Observations:                   44
Model:                            GLM   Df Residuals:                       42
Model Family:                Gaussian   Df Model:                            1
Link Function:               identity   Scale:                      0.00026237
Method:                          IRLS   Log-Likelihood:                 120.00
Date:                Wed, 28 Oct 2020   Deviance:                     0.011019
Time:                        11:31:04   Pearson chi2:                   0.0110
No. Iterations:                     3
Covariance Type:            nonrobust
==============================================================================
                 coef    std err          z      P>|z|      [0.025      0.975]
------------------------------------------------------------------------------
x1             0.0064      0.002      2.612      0.009       0.002       0.011
const          0.1379      0.002     56.414      0.000       0.133       0.143
==============================================================================
T-values:  [ 2.61229786 56.41381163]
```

(b) Scenario 2: “unlearning” data with a simple two group difference design matrix

Scenario 3:

```
                 Generalized Linear Model Regression Results
==============================================================================
Dep. Variable:                      y   No. Observations:                   44
Model:                            GLM   Df Residuals:                       42
Model Family:                Gaussian   Df Model:                            1
Link Function:               identity   Scale:                       0.0057920
Method:                          IRLS   Log-Likelihood:                 51.918
Date:                Wed, 28 Oct 2020   Deviance:                      0.24326
Time:                        11:33:15   Pearson chi2:                    0.243
No. Iterations:                     3
Covariance Type:            nonrobust
==============================================================================
                 coef    std err          z      P>|z|      [0.025      0.975]
------------------------------------------------------------------------------
x1            -0.0037      0.011     -0.325      0.745      -0.026       0.019
const          0.2156      0.011     18.773      0.000       0.193       0.238
==============================================================================
T-values:  [-0.32520816 18.77344661]
```

(c) Scenario 3: base case of “normal” data and simple design matrix

Figure 19: GLM results for the three explored scenarios.
